# Screening in Human Genetics – new opportunities, new responsibilities

**DOI:** 10.1515/medgen-2026-3026

**Published:** 2026-11-06

**Authors:** Christian P. Schaaf, Sabine Rudnik-Schöneborn

**Affiliations:** Heidelberg University Institute of Human Genetics Im Neuenheimer Feld 366 69120 Heidelberg Germany; Medical University Innsbruck Institute of Human Genetics Innsbruck Austria

Only a generation ago, genetic testing was almost exclusively performed for specific clinical indications: an affected individual, a suggestive family history, or prenatal findings requiring further clarification. In this setting, human genetics primarily served as a diagnostic discipline, helping to establish disease causes, define the chance of recurrence, and support clinical management. Today, genetic analyses are increasingly offered to individuals with the aim of identifying disease predisposition before clinical manifestations occur. This development has been driven not only by rapid advances in sequencing technologies and declining costs, but equally by the growing number of genetic conditions for which early diagnosis enables meaningful prevention, surveillance or treatment. As a consequence, screening has become one of the defining applications of modern human genetics, expanding the role of our discipline from diagnosis towards preventive medicine.

## Moving from diagnosis towards prevention

The technical progress of the past two decades has been remarkable. Next-generation sequencing has fundamentally changed genetic diagnostics, allowing simultaneous analysis of large numbers of genes – all the way to exomes and genomes – with unprecedented speed and at declining cost. However, technology alone does not explain why screening has become such a prominent topic in medical genetics. A screening program is justified not because a test is technically feasible, but because the identification of disease risk before the onset of symptoms leads to better health outcomes.

The decisive change has therefore been the increasing clinical actionability of genetic diagnoses. Individuals with tumor disposition syndromes benefit from intensified surveillance or prophylactic interventions, children with familial hypercholesterolemia from early lipid-lowering therapy, and patients with an expanding number of rare genetic disorders from treatments that are most effective before irreversible organ damage has occurred [1, 2]. Even where curative therapies are unavailable, surveillance, anticipatory guidance and informed reproductive decision-making may improve outcomes for patients and their families.

These developments invite a re-examination of the principles that have guided population screening for decades. The criteria proposed by Wilson and Jungner in 1968 [3] remain highly relevant and have recently been refined for the genomic era [4]. The availability of a sensitive genetic test alone is not sufficient to justify screening. Screened conditions should represent an important health problem and, crucially, offer interventions that improve outcomes when initiated early. Genomic technologies have greatly expanded the range of detectable conditions, but they have not changed these fundamental principles. The central question is therefore no longer whether we can identify individuals at risk, but which conditions should be screened for, at what stage of life, and under which medical, ethical and legal conditions [5].

The five articles assembled in this issue approach screening from different perspectives. Read together, however, they tell a common story: how human genetics is increasingly moving from the diagnosis of inherited disease towards its prevention.

## Screening across the life course

Screening approaches today cover the entire life span – from preconception all the way into adulthood – with each stage presenting distinct objectives, opportunities and challenges.

The earliest opportunity for screening arises before pregnancy. Reproductive carrier screening addresses healthy individuals or couples without a known family history of a specific disorder and aims to identify an increased risk of having a child affected by an autosomal recessive or X-linked condition. Unlike most other screening programs discussed in this issue, its primary objective is not the health of the individual being tested, but information that enables informed reproductive decisions. It is estimated that about 4–5 % of Non-Finnish European couples are at increased risk for children with a severe disease. Broad carrier panels raise questions regarding disease selection, residual risk, informed consent, and access to high-quality genetic counselling. For a possible implementation in Germany, the legal framework, genetic, reproductive, and ethical perspectives have to be considered (Rudnik-Schöneborn et al., this issue).

During pregnancy, non-invasive prenatal testing (NIPT) based on cell-free fetal DNA in maternal blood has fundamentally changed screening for common fetal aneuploidies. Its rapid technical development also illustrates an important principle extending far beyond prenatal medicine: technological possibilities tend to evolve more rapidly than clinical indications. Targeted analysis of cell-free fetal DNA can already enable non-invasive prenatal diagnosis of selected monogenic disorders, although its clinical use is currently restricted to specific conditions and risk constellations. By contrast, comprehensive genome-wide detection of single-nucleotide variants from cell-free fetal DNA remains investigational (Weise and Liehr, this issue).

Conventional newborn screening has demonstrated for decades that early identification of treatable disorders can prevent severe morbidity and mortality. Genomic newborn screening builds upon this experience while expanding the spectrum beyond conditions detectable through biochemical markers. This raises questions of disease selection, actionability, variant interpretation, management of uncertain findings, and long-term follow-up. Genomic newborn screening should therefore not be regarded simply as the application of sequencing technologies to newborns, but as the careful extension of a highly successful public health program into the genomic era (Hindricks et al., this issue).

Familial hypercholesterolemia illustrates why genetic screening may also play a greater role during childhood. As one of the most common monogenic disorders, it fulfils many classical screening criteria: it is relatively frequent, can be identified before symptoms occur, and early treatment substantially reduces long-term cardiovascular risk. Identifying an affected child can additionally lead to cascade testing and diagnosis of previously unrecognized relatives, demonstrating that the benefits of genetic screening often extend beyond the individual tested (Piel-Behrendt and Schlein, this issue).

In adulthood, population-based screening might identify individuals with hereditary cancer predisposition, enabling surveillance, preventive interventions, and cascade testing. In current practice, a tumor disposition syndrome is usually identified only if personal- or family-history-based clinical criteria are met. Top candidates for a population-based genomic screening are the two most frequent cancer syndromes, i.e. hereditary breast and ovarian cancer, and Lynch syndrome. At the same time, moving from clinically affected families to unselected populations creates new challenges. Penetrance estimates derived from selected clinical cohorts cannot simply be transferred to the general population, and risk communication must account for genetic, familial, and environmental modifiers (Grohs et al., this issue).

Across these applications, the fundamental questions remain similar: Which conditions justify screening? When does knowledge provide measurable benefit? And how can genomic information be translated into improved health while respecting individual autonomy and avoiding unnecessary harm?

## Responsible implementation: the German perspective

Germany has approached the expansion of genetic screening somewhat differently from many other countries. While much of the international discussion has focused on the technical feasibility and clinical implementation, the German debate has been equally shaped by the question of how genetic information should be generated, communicated and used responsibly.

The Genetic Diagnostics Act (Gendiagnostikgesetz, GenDG), which came into force in 2010, remains the central legal framework governing genetic testing for medical purposes in Germany. It recognizes that genetic information is often predictive, may have implications for biologically related family members, and can remain relevant throughout an individual’s lifetime. The Act therefore emphasizes informed consent, protection against genetic discrimination, quality assurance, and the individual’s right not to know [6]. For predictive testing, it also requires qualified genetic counselling, with recommendations of the Gendiagnostik-Kommission (GEKO) translating these principles into practical standards [7]. While such requirements may sometimes be perceived as obstacles to implementation, they can equally be understood as safeguards promoting trust in genetic medicine.

The German framework also distinguishes prevention from reproductive medicine. Reproductive carrier screening and prenatal screening for genetic conditions, such as non-invasive prenatal testing (NIPT), raise issues concerning the selection of the health-related characteristics in a child and are therefore subject to additional legal and ethical considerations.

Population-based sequencing, genomic newborn screening, and increasingly comprehensive reproductive screening programs were not yet fully conceivable when the GenDG was drafted. Nevertheless, its underlying principles have lost little of their relevance [8]. If anything, the expansion of genetic screening has reinforced their importance. As genetic information becomes available to growing numbers of healthy individuals, careful counselling, proportionality, respect for individual autonomy, and the right not to know become more – not less – important.

## Opportunities and responsibilities

In genomic screening, opportunities and responsibilities are directly connected. The relevant question is rarely whether a screening program is technically feasible, but whether its expected benefit justifies the resources required and the potential consequences for individuals, families, and healthcare systems.

Moving genetic testing from clinically selected families into population screening changes the context in which genetic findings are interpreted. Penetrance and disease expression may differ from estimates derived from highly selected cohorts with strong personal or family histories. Similarly, genomic medicine may technically allow testing for hundreds or thousands of conditions, but the success of screening does not depend on the number of conditions included. It relies on careful disease selection, robust evidence for clinical benefit and well-defined pathways for confirmatory testing and treatment [9].

Equitable implementation is equally important. Population screening can achieve its public health objectives only if access is independent of socioeconomic status, educational background or geographical location. Genomic databases and risk prediction models must adequately represent diverse populations, and healthcare systems must provide access to counselling, confirmatory diagnostics, preventive interventions, and long-term follow-up.

Every new opportunity created by genetic screening is accompanied by a corresponding responsibility: to define appropriate target conditions, to generate robust evidence, to communicate risk transparently, to respect individual autonomy, and to continuously evaluate clinical benefit. These responsibilities are not obstacles to innovation, but prerequisites for translating the expanding possibilities of genetic screening into meaningful improvements in patient care.

## Conclusion

The contributions assembled in this issue illustrate how broadly the concept of genetic screening has expanded – from preconception to adulthood – and how it is becoming a defining application of modern human genetics. Its success, however, cannot be measured by technological progress alone. New screening programs require evidence of clinical benefit, appropriate selection of target conditions, transparent communication of benefits and limitations, high-quality genetic counselling, and equitable access to follow-up care. Realizing the full potential of genomic screening will depend on careful implementation of programs that are scientifically sound, clinically meaningful, and socially responsible. Balancing scientific innovation with careful reflection on its consequences will remain a central responsibility of human genetics in the genomic era.
